# Supplementary figures and images for: RNA-Seq and ATAC-Seq Reveal CYP26A1-Mediated Regulation of Retinoic Acid-Induced Meiosis in Chicken Primordial Germ Cells
Source: Animals (Basel). 2024 Dec 25;15(1):23. doi: 10.3390/ani15010023 (PMC11718974; doi:10.3390/ani15010023)

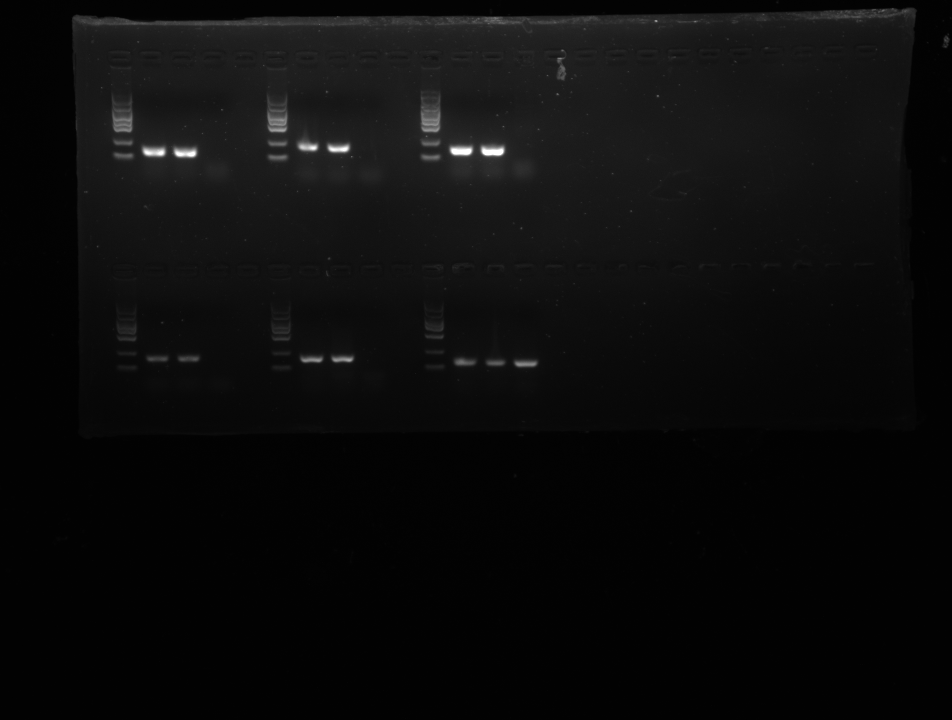

Supplement: Supplementary file 1 [file animals-15-00023-s001.zip › Figure S1.tif]
